# Hard shell, soft core? Multi-disciplinary and multi-national insights into mental toughness among surgeons

**DOI:** 10.3389/fsurg.2024.1361406

**Published:** 2024-04-05

**Authors:** Leonard Knoedler, Jillian Dean, Samuel Knoedler, Martin Kauke-Navarro, Katharina Hollmann, Michael Alfertshofer, Sabrina Helm, Lukas Prantl, Rainer Schliermann

**Affiliations:** ^1^ Division of Plastic Surgery, Department of Surgery, Yale School of Medicine, New Haven, CT, United States; ^2^ Department of Plastic, Hand and Reconstructive Surgery, University Hospital Regensburg, Regensburg, Germany; ^3^ University of Pittsburgh School of Medicine, Pittsburgh, PA, United States; ^4^ Division of Hand, Plastic and Aesthetic Surgery, Ludwig-Maximilians-University Munich, Munich, Germany; ^5^ Department of Oromaxillofacial Surgery, Ludwig-Maximilians-University Munich, Munich, Germany; ^6^ Faculty of Social and Health Care Sciences, Regensburg University of Applied Sciences, Regensburg, Germany

**Keywords:** mental toughness, mental health, resilience, robustness, psychology, surgery

## Abstract

**Background:**

With the prevalence of burnout among surgeons posing a significant threat to healthcare outcomes, the mental toughness of medical professionals has come to the fore. Mental toughness is pivotal for surgical performance and patient safety, yet research into its dynamics within a global and multi-specialty context remains scarce. This study aims to elucidate the factors contributing to mental toughness among surgeons and to understand how it correlates with surgical outcomes and personal well-being.

**Methods:**

Utilizing a cross-sectional design, this study surveyed 104 surgeons from English and German-speaking countries using the Mental Toughness Questionnaire (MTQ-18) along with additional queries about their surgical practice and general life satisfaction. Descriptive and inferential statistical analyses were applied to investigate the variations in mental toughness across different surgical domains and its correlation with professional and personal factors.

**Results:**

The study found a statistically significant higher level of mental toughness in micro-surgeons compared to macro-surgeons and a positive correlation between mental toughness and surgeons' intent to continue their careers. A strong association was also observed between general life satisfaction and mental toughness. No significant correlations were found between the application of psychological skills and mental toughness.

**Conclusion:**

Mental toughness varies significantly among surgeons from different specialties and is influenced by professional dedication and personal life satisfaction. These findings suggest the need for targeted interventions to foster mental toughness in the surgical community, potentially enhancing surgical performance and reducing burnout. Future research should continue to explore these correlations, with an emphasis on longitudinal data and the development of resilience-building programs.

## Introduction

The medical profession, characterized by its high-stress environment and life-or-death decisions, places extraordinary psychological demands on physicians and surgeons. The imperative to maintain mental well-being within this cohort is of utmost importance, yet it is a challenge that has only recently begun to garner the attention it deserves. Recent studies, including those by Jesuyajolu and colleagues in (1), highlights a significant incidence of burnout among physicians and surgeons, underscoring the urgent need to prioritize their mental health (1). Physicians, who may be as much as 2.45 times more likely to commit suicide than non-physicians (2), face an alarming 47% prevalence of burnout symptoms among surgeons, with variations observed across different surgical specialties (1). Despite the growing recognition of this issue, comprehensive research that penetrates the nuanced dimensions of mental health in the surgical profession remains scarce.

Here, we operationalize mental toughness as a multi-faceted psychological attribute that enables surgeons to perform consistently under varied degrees of stress and pressure. This construct is distinct yet related to resilience, hardiness, and grit, each of which contributes uniquely to the psychological profile of medical professionals. Resilience, as described in the work of Roslan et al. (3), refers to the dynamic process of positive adaptation in response to significant adversity. It is a construct that evolves through interaction with environmental challenges and is characterized by an individual's capacity for rebound and growth following disruptions. Hardiness, closely related to resilience, involves a dispositional resilience that encompasses commitment, control, and challenge. It is often considered a pathway through which resilience can be developed, particularly in response to ongoing adversities. Mental toughness, while sharing aspects of resilience and hardiness, is particularly salient in high-performance environments such as surgery. It is characterized by an unyielding self-belief and a desire to thrive, not just survive, amidst adversity. Unlike resilience, which can imply a return to a prior state of equilibrium, mental toughness suggests an enhanced state of functioning that maximizes performance potential, also described by Anton & Stefanidis (4). Grit, defined by perseverance and passion for long-term goals, is associated with success over prolonged periods. While it includes an aspect of resilience, grit is distinct in its focus on long-term persistence towards goals rather than the immediate bounce-back from adversity (3). In the context of our study, mental toughness in surgeons is not just the ability to recover from challenges but also the sustained application of resilience, hardiness, engagement, and grit to their demanding professional lives. This understanding of mental toughness as a composite of these related but distinct constructs adds depth to our exploration of psychological profiles within the surgical profession.

Unlike more tangible medical outcomes, mental toughness is an abstract construct, difficult to quantify and influenced by a myriad of individual, professional, and cultural factors (5, 6). However, as Percy et al. have indicated, there is a lack of formal evaluation or development programs focusing on the psychological skills necessary for cultivating such toughness in the surgical profession. The challenge lies not only in defining and measuring this construct but also in accounting for the diverse settings and specialties within the surgical field. Furthermore, research in this area is impeded by the paucity of validated instruments tailored to the unique pressures of surgical practice (7), as well as by potential resistance within the medical community to acknowledge and discuss issues of mental health (8). This omission has far-reaching implications, not only for the surgeons' own health but also for patient safety and the overall quality of care (5).

The association between physician burnout and patient safety is well-established, indicating that burnout contributes significantly to medical errors (9). Moreover, increased rates of burnout among surgeons are linked to a higher frequency of medical malpractice lawsuits, with an average cost of $371,054 per case (10, 11). Thus, enhancing mental toughness in physicians and surgeons could have a considerable impact on reducing the incidence of errors and improving patient outcomes (12).

Despite a growing body of literature addressing various facets of surgeons' mental health, including burnout (13), suicidal ideation (2, 14), and mental workload, there is a paucity of research specifically focused on mental toughness in a comprehensive, multinational context. This study aims to bridge this gap by evaluating mental toughness within a diverse cohort of surgeons representing various specialties, offering insights into the status of surgeons' mental toughness, the factors that impact it, and potential strategies for enhancement. Given the limited prior research in the realm of mental toughness and its related determinants in the surgical field, our approach is exploratory, focused on addressing broad research questions rather than testing specific hypotheses.

The central questions we seek to answer are as follows: (1) Are there statistically significant and meaningful variations in mental toughness among surgeons related to surgical variables? (2) Are there statistically significant and meaningful differences in mental toughness among surgeons related to non-surgical variables? (3) Are there discernible correlations between mental toughness and general life satisfaction, as well as the application of selected psychological techniques, respectively? Through this inquiry, we aspire to contribute to the enhancement of patient care by prioritizing the well-being of physicians. This research initiative is guided by the need to support physicians and surgeons in developing the mental toughness necessary to thrive in their demanding profession.

## Methods

This study employed a cross-sectional design. A non-probabilistic convenience sample was composed of surgeons from both English-speaking regions (North America, South Africa, Europe) and German-speaking countries (Germany, Switzerland, Austria). The survey was disseminated through a variety of channels, including email mailing lists and newsletters of various professional surgical societies, which were informed about the study's objectives and potential contributions to better understand its context. Subsequently, an internet link was distributed to access the online questionnaire. The questionnaire itself was designed and administered through the SoSci Survey tool (www.soscisurvey.de). Data collection occurred in July 2022, with detailed sample characteristics presented in the results section. In total, 104 surgeons successfully completed the questionnaire.

The online questionnaire comprised three main sections: (a) Mental Toughness Assessment: The questionnaire featured an 18-item short version of the Mental Toughness Questionnaire (MTQ-18) in its original English form (15) and a rigorously validated and psychometrically tested German version (16). The MTQ-18 operationalizes mental toughness through four interrelated components: challenge (e.g., “I generally cope well with any problems that occur”), commitment (e.g., “I usually find it hard to summon enthusiasm for the tasks I have to do”), control (e.g., “I generally feel in control” for control-life and “Even when under considerable pressure, I usually remain calm” for control-emotion), and confidence (e.g., “However bad things are, I usually feel they will work out positively in the end” for confidence-ability and “If I feel somebody is wrong, I am not afraid to argue with them” for confidence-interpersonal). Respondents provided their answers using a 5-point Likert-type scale ranging from 1 (“strongly disagree”) to 5 (“strongly agree”). To assess mental toughness, a sum score was calculated. Reliability analyses indicated good internal consistencies for both the English version (Cronbach's *α* = 0.81) and the German version (*α* = 0.83), with an overall sample reliability of *α* = 0.82. These results justified the parallel application of both versions for cross-cultural analyses.

(b) Background Information: The questionnaire further explored the complexity of surgical procedures by categorizing the surgeons into two groups based on their practice scope. “Micro surgery” was defined to include specialized fields such as plastic surgery, oral and maxillofacial surgery, spinal surgery, and neurosurgery, which often involve intricate and detailed operative techniques. In contrast, “Macro surgery” encompassed all other surgical specialties. This distinction was used to assess and compare the difficulty of operations across the two categories. The first item examined whether the primary objective of the surgeons' procedures was short- or medium-term life-saving, focusing on critical conditions like heart disease, lung disease, or cancer. The second item sought information on whether surgeons primarily treated patients with general diseases, asking them to gauge the severity of these conditions from mild to severe and life-threatening, where surgery is imperative for patient survival.

Additionally, it inquired about psychological skills and techniques relevant to both sports and medical performance, including imagery, relaxation, activation, and behavior routines. These questions drew from previous research (17–20). (c) General Life Satisfaction Assessment: Finally, a single-item short-scale for assessing general life satisfaction [L-1 (21, 22);] was administered. Respondents were asked to rate their overall life satisfaction with a question that included a scale from 0 (“not at all satisfied”) to 10 (“completely satisfied”). The scale is available in both German (21) and English (22), which demonstrated good reliability and evidence of validity in both versions, justifying its parallel use in our study.

Following a comprehensive examination of the dataset for the identification of outliers and the verification of accurate data entry procedures, a series of statistical analyses were carried out, encompassing both descriptive and inferential methodologies. Descriptive statistics were computed to furnish a concise summary of the dataset, encompassing relevant measures such as means, standard deviations, and frequencies, as deemed suitable for the data under examination. The inferential phase of analysis entailed the utilization of various statistical techniques to explore relationships and discrepancies within the dataset. Specifically, one-way Analysis of Variance (ANOVA) tests were executed, and Pearson's bivariate correlations were calculated. The normal distribution assumption was evaluated using histograms and the Shapiro–Wilk test, and homogeneity of variances between compared groups was tested via Levene-test. Unless otherwise specified, these assumptions were ascertained to hold. The delineation of statistically significant results was defined with a significance threshold of *p* ≤ 0.05. The evaluation of the magnitude of differences or effects adhered to Cohen's (23) established guidelines, where small effects were represented by *η*^2^ = 0.01, medium effects by *η*^2^ = 0.06, and large effects by *η*^2^ = 0.14. Interpretation of Pearson's correlations (*r*) followed the parameters of small correlations denoted by *r* = 0.10, medium correlations indicated by *r* = 0.30, and large correlations represented by *r* = 0.50.

## Results

In this multinational study, a sample comprising 104 surgeons was examined. Among these, 54 surgeons hailed from English-speaking countries (North America, Europe, and South Africa), while 50 surgeons originated from German-speaking countries (Germany, Switzerland, Austria). The average clinical experience among the participants was 16.89 ± 12.85 years. Notably, most of the surgeons (*n* = 52/99; 53%) held leadership positions, and the mean number of surgeries performed per week was 7.79 ± 5.93 (SD). Further demographic data is shown in Table 1.

**Table 1:** Characterization of the study population.

Total participants (Frequency in %)	104	
English speaking countries	54	52%
German speaking countries	50	48%
Position in hospital (Frequency in %)		
Chief physician	11	11.1%
Chief senior physician	14	14.1%
Senior physician	27	27.3%
Specialist	19	19.2%
Resident	23	23.2%
Others	5	5.1%
Location of surgical training (Frequency in %)		
Europe	51	51.5%
North America	26	26.3%
South Africa	22	22.2%
Surgical experience in years (SD)	16.89	12.85
Operations per week (SD)	7.79	5.93
Operation durations in hours (SD)	2.59	1.62
Primary goal to save patient lives? (Frequency in %)		
Yes	81	87.1%
No	12	12.9%
Applied psychological techniques prior to operation (SD)	2.23	1.13
Surgical role model		
Yes	70	79.5%
No	18	20.5%
Pursue surgical career again? (Frequency in %)		
Yes	63	71.6%
No	25	28.4%
Mental toughness (SD)	3.61	0.52
General life-satisfaction (SD)	7.44	1.96

The results of our analysis indicated that there were no statistically significant differences in mental toughness based on surgical specialty [pediatric vs. non-pediatric; *F* (1.95) = 1.26; *p* = .264; *η*^2^ = .013], professional position within the hospital [leading vs. non-leading; F (1.91) = .61; *p* = .437; *η*^2^ = .007], years of experience as a surgeon [≤7 vs. >22 years; *F* (1.59) = .38; *p* = .539; *η*^2^ = .006], the number of surgeries performed per week [≤5 vs. >8; *F* (1.68) = .02; *p* = .897; *η*2 ≤ .001], the duration of surgeries [≤2 vs. >3 h; *F* (1.71) = 1.60; *p* = .211; *η*^2^ = .022], or the nature of surgeries categorized by general illnesses [mild vs. severe vs. very severe; *F* (2.74) = .25; *p* = .782; *η*^2^ = .007]. However, a statistically significant difference of medium magnitude was observed when comparing micro-surgery to macro-surgery [*F* (1.95) = 5.73; *p* = .019; *η*^2^ = .057], with micro-surgeons displaying higher levels of mental toughness. Additionally, there was a notable trend towards statistically significant lower mental toughness in the general surgery category when compared to other surgical specialties [*F* (1.95) = 2.78; *p* = .099; *η*^2^ = .028].

Participants' intentions to continue pursuing their current career path exhibited a medium-sized impact on their mental toughness [*F* (1.85) = 10.58; *p* = .002; *η*^2 ^= .111], with those intending to continue on in their careers showing higher mental toughness. A mild correlation emerged between general life satisfaction and mental toughness (*r* = 0.382, *p* < .0001, *n* = 87). Our analysis revealed statistically significant medium-sized differences in mental toughness among surgeons from different countries of surgical training [North America vs. Europe vs. South Africa; *F* (2.94) = 3.13; *p* = .048; *η*^2^ = .062], with higher mental toughness values observed among surgeons trained in North America and Europe (Table 2; Post-Hoc tests: LSD test: North America & Europe >South Africa: *p* = .027/.028; Bonferroni test: *p* = .080/.083). Lastly, correlations between the application of psychological techniques (imagery; relaxation; activation; behavior routines) and mental toughness were not statistically significant (*r* = −.102; *p* = .341; *n* = 90).

**Table 2:** Descriptive statistics for the subgroup analyses.

Subgroup	*n*	Mean	SD
Pediatric surgeons	22	3.53	0.49
Leading position in hospital	51	3.68	0.47
Low experienced surgeon	30	3.58	0.63
Less operations per week	40	3.60	0.47
Short operations	53	3.62	0.47
Mild illnesses	21	3.62	0.64
Micro surgery	24	3.85	0.51
General surgery	28	3.50	0.54
Pursuing the same career path	62	3.77	0.44
Surgical training in South Africa	22	3.40	0.57
Non-pediatric surgeons	75	3.67	0.52
Non-leading position in hospital	42	3.59	0.57
Many years experienced surgeon	31	3.66	0.37
Many operations per week	30	3.59	0.60
Long operations	20	3.78	0.50
Severe illness	30	3.72	0.50
Very severe illnesses	26	3.70	0.41
Macro surgery	73	3.57	0.50
Other specialties	69	3.69	0.50
Not pursuing the same career path	25	3.40	0.57
	25	3.74	0.42
Surgical training in Europe/North America/Europe	50	3.69	0.51

N, sample size subgroup; SD, standard deviation; subgroup definitions: professional position in hospital [leading position in hospital (chief physician; Chief Senior Physician; Senior Physician) vs. non-leading position (specialist; resident)]; experience as a surgeon [jeweils 1. & 3. 33% der Werteverteilung; low experienced surgeon (≤7.3 years) vs. high experienced surgeon (>20 years)]; operations per week [jeweils 1. & 3. 33% der Werteverteilung; less operations per week (≤5) vs. many operations per week (>8)]; duration of the operations [jeweils 1. 33% & 3. 33% der Werteverteilung; short operations (≤2 h) vs. long operations (>3 h)]; operations with patients of general diseases [mild general illnesses vs. severe general illnesses (patients that poses a constant threat to life) vs. very severe (patients who would probably not survive without these operations)]; ÜBERBEGRIFF?!! [micro surgery (xxxx) vs. macro surgery (xxxx)].

## Discussion

The initial phase of our research has provided a systematic exploration into the construct of mental toughness within the surgical profession, a concept frequently discussed in psychological literature but less frequently examined in medical contexts. The construct of mental toughness encompasses a spectrum of psychological resources, including but not limited to resilience, cognitive flexibility, and stress tolerance, which are crucial for optimal performance under the demanding conditions of surgical practice. This study operationalizes mental toughness as it pertains specifically to the surgical domain, investigating its manifestation across various surgical specialties and its correlation with performance outcomes.

The variance in mental toughness noted across different surgical domains, and in particular between micro-surgeons and macro-surgeons, invites a discussion on the relationship between the nature of surgical work and psychological resilience. While it may be posited that the specific demands of microsurgery, which often include highly technical and prolonged procedures, could foster greater mental toughness, an alternative interpretation is also plausible. It is conceivable that individuals who inherently possess higher levels of mental toughness are more inclined to pursue the field of microsurgery. This latter explanation suggests a self-selection bias where the characteristics of the surgeon, rather than the demands of the specialty, are responsible for the observed differences in mental toughness. Given the cross-sectional design of our study, we cannot determine the direction of this association. Future research could benefit from longitudinal designs to ascertain whether mental toughness is a pre-existing trait that influences the choice of surgical specialty or an attribute developed through the unique challenges of a surgeon's chosen field.

Moreover, the interplay between mental toughness and the broader concept of surgical efficacy, as inferred from our research, could be influenced by a variety of factors. These include individual coping mechanisms, the inherent complexity of specific surgical procedures, and the aggregate impact of a surgeon's experience. Our study conceptualizes “surgical efficacy” as the surgeon's ability to navigate the demanding operative environment effectively, maintain focus under pressure, and make critical decisions with precision. While we did not directly measure clinical outcomes such as postoperative complications, surgery duration, or patient recovery times, our use of “surgical efficacy” refers to the cognitive and psychological components of performing surgery. This nuanced understanding of mental toughness highlights the potential for tailored interventions to enhance this attribute in surgical trainees and professionals.

While our results have provided significant insights into the profiles of mental toughness among surgeons, they also prompt further investigation into the development of targeted strategies to bolster this attribute within the surgical profession. Such strategies could be instrumental in not only improving technical performance but also in enhancing the overall well-being of surgeons, thereby potentially reducing the incidence of burnout and improving patient care outcomes. The implications of our findings for surgical education and practice are substantial and warrant a multidisciplinary approach to further inquiry and application.

One of the most noteworthy outcomes of our study is the revelation of substantial disparities in mental toughness when contrasting micro-surgery against macro-surgery, as well as general surgery against other specialized domains within the field. Specifically, micro-surgeons exhibited markedly heightened levels of mental toughness in comparison to their counterparts in macro-surgery. This discovery aligns with the work of Arora et al. (24), who examined the impact of stress on surgical performance. Their systematic review underscored the detrimental effects of heightened stress levels, particularly in laparoscopic procedures, where stress was shown to correlate with subpar technical performance. Our observation that micro-surgeons exhibit greater mental toughness could provide a compelling explanation for their superior performance, suggesting that mental resilience plays a pivotal role in surgical success.

Our study identified a notable correlation between surgeons' career intentions and their levels of mental toughness. Specifically, those surgeons with a strong expressed intent to continue in their surgical careers were found to have higher levels of mental toughness. This correlation echoes the findings of Percy et al. (5), which found that staff surgeons tend to exhibit greater mental toughness compared to residents, suggesting an association between surgical experience and mental toughness. Similarly, Wetzel et al. (25) observed that senior surgeons often possess advanced coping strategies. These findings may suggest a relationship where experience contributes to mental toughness or vice versa; however, given the cross-sectional nature of our study, we cannot infer causality. Thus, while a positive correlation between surgical experience and mental toughness is evident, further research is required to understand the directionality and underlying mechanisms of this relationship.

While exploring the relationship between psychological skills and techniques and mental toughness, we encountered an unexpected outcome of no statistically significant correlations. However, a strong correlation was established between general life satisfaction and mental toughness. This result echoes the work of Shanafelt et al. (26), who emphasized the impact of burnout on surgeons' career satisfaction. The findings of our study imply that overall life satisfaction and mental toughness are engaged in a reciprocal relationship, each playing a pivotal role in reinforcing the other. This circular relationship underscores the complex interplay between personal satisfaction and professional resilience, highlighting how a surgeon's well-being is dynamically influenced by factors both inside and outside the operating room.

This study presents several limitations that should be considered when interpreting the results. One primary concern is the use of a non-probabilistic convenience sample of 104 participants. While this number provided a basis for our exploratory analysis, it is recognized that such a sample size may limit the statistical power of the study, increasing the risk of Type II errors where true differences or effects might not be detected. Moreover, convenience sampling does not ensure a representative distribution of the target population, which could introduce bias and limit the generalizability of the findings. Due to the distribution method of our online questionnaire, which was disseminated via various email mailing lists and newsletters of multiple professional surgical societies, it is not feasible to accurately determine the total number of surgeons contacted. Consequently, this may introduce a potential selection bias, as it is unclear whether the respondents are representative of the broader surgical community. Another limitation is that the survey's design did not include a query for gender, which was a deliberate choice to maintain the anonymity of respondents and to focus on psychological attributes without preconceived notions of gender influence. We acknowledge that this decision may overlook potential gender-specific insights into mental toughness in the surgical field.

In terms of the findings, while a statistically significant difference was observed in mental toughness between micro-surgeons and macro-surgeons, the medium magnitude of this effect (*η*^2 ^=^ ^.057) and the mild correlation reported (*r* = 0.382) warrant cautious interpretation. Although statistically significant, these results reflect a modest relationship that must be considered in the context of the study's sampling and methodological constraints. It is also important to note that the cross-sectional design of the study restricts the ability to infer causality from the observed associations. Longitudinal studies would be necessary to draw stronger inferences about the development and impact of mental toughness over time within the surgical profession. Lastly, the use of self-report measures for assessing mental toughness and other psychological constructs, while practical and widely accepted, can be susceptible to response biases such as social desirability or inaccurate self-assessment. This could have influenced the accuracy of the data collected, particularly concerning the self-reported application of psychological skills and techniques.

This multinational and multidisciplinary study has provided invaluable insights into the psychological dimensions of surgical practice. It underscores the paramount importance of mental toughness, particularly in the context of micro-surgery and career intentions, while shedding light on its potential impact on surgical performance. However, the influence of individual career intentions and overall life satisfaction on mental toughness should be considered. Strategies aimed at enhancing mental toughness should be tailored to the unique demands of different surgical specialties and the holistic well-being of surgeons. Addressing these multifaceted aspects can contribute to improved surgeon well-being and patient care. Future research should explore interventions designed to enhance mental toughness and mitigate the effects of stress in surgical practice, fostering a more resilient surgical community.

## Conclusion

This study highlighted the significance of mental toughness as a multifaceted psychological construct with the potential to influence surgical performance and outcomes. The variance observed in mental toughness across different surgical specialties suggests that the nature of surgical work may play a role in shaping this attribute. The association between a surgeon's career commitment and their mental toughness invites further reflection on how professional dedication can impact psychological resilience. Moreover, the link between life satisfaction and mental toughness underscores the complex interplay between a surgeon's personal well-being and their professional capacity to withstand stress.

While our findings offer valuable insights, they also illuminate the challenges inherent in studying such an intricate construct within a diverse professional population. Future research should seek to expand upon these initial findings through longitudinal studies and larger, more representative samples. In doing so, the surgical community can better understand the development of mental toughness and its implications for practice. The study's implications extend to surgical education and professional development, where there is a clear opportunity to incorporate strategies for building mental toughness. Addressing both the personal and professional dimensions of surgeons' lives, such interventions could contribute to reducing burnout and enhancing patient care. Our research adds to the growing recognition of the importance of psychological well-being in the medical profession and calls for a continued, concerted effort to support surgeons in cultivating the resilience necessary to navigate the demanding nature of their work.

## Data Availability

The original contributions presented in the study are included in the article/Supplementary Material, further inquiries can be directed to the corresponding authors.
